# A unified approach to molecular epidemiology investigations: tools and patterns in California as a case study for endemic shigellosis

**DOI:** 10.1186/1471-2334-9-184

**Published:** 2009-11-24

**Authors:** Sawsan Al-Nimri, Woutrina A Miller, Barbara A Byrne, Gerry Guibert, Lily Chen

**Affiliations:** 1 Biology Department, San Francisco State University, 1600 Holloway Ave, San Francisco, CA, 94132, USA; 2 Pathology, Microbiology & Immunology Department, School of Veterinary Medicine, University of California, Davis, CA, 95616, USA; 3 Monterey County Public Health Department, 1270 Natividad Rd, Salinas, CA, 93906, USA

## Abstract

**Background:**

Shigellosis causes diarrheal disease in humans from both developed and developing countries, and multi-drug resistance is an emerging problem. The objective of this study is to present a unified approach that can be used to characterize endemic and outbreak patterns of shigellosis using use a suite of epidemiologic and molecular techniques. The approach is applied to a California case study example of endemic shigellosis at the population level.

**Methods:**

Epidemiologic patterns were evaluated with respect to demographics, multi-drug resistance, antimicrobial resistance genes, plasmid profiles, and pulsed-field gel electrophoresis (PFGE) fingerprints for the 43 *Shigella *isolates obtained by the Monterey region health departments over the two year period from 2004-2005.

**Results:**

The traditional epidemiologic as well as molecular epidemiologic findings were consistent with endemic as compared to outbreak shigellosis in this population. A steady low level of cases was observed throughout the study period and high diversity was observed among strains. In contrast to most studies in developed countries, the predominant species was *Shigella flexneri *(51%) followed closely by *S. sonnei *(49%). Over 95% of *Shigella *isolates were fully resistant to three or more antimicrobial drug subclasses, and 38% of isolates were resistant to five or more subclasses. More than half of *Shigella *strains tested carried the *tetB*, *catA*, or *bla*_TEM _genes for antimicrobial resistance to tetracycline, chloramphenicol, and ampicillin, respectively.

**Conclusion:**

This study shows how epidemiologic patterns at the host and bacterial population levels can be used to investigate endemic as compared to outbreak patterns of shigellosis in a community. Information gathered as part of such investigations will be instrumental in identifying emerging antimicrobial resistance, for developing treatment guidelines appropriate for that community, and to provide baseline data with which to compare outbreak strains in the future.

## Background

Shigellosis, commonly known as acute bacillary dysentery, is a global human health problem, with over 165 million cases and 1 million deaths occurring each year [1]. *Shigella sonnei *has been reported as the most prevalent endemic species in developed countries, *S. flexneri *as the most prevalent endemic species in developing countries, and *S. dysenteriae *is known to cause sporadic outbreaks and epidemics worldwide [1-5]. In the United States, an estimated 450,000 people are infected each year, with most clinical cases occurring in children [1,5,6].

Although shigellosis is generally a self-limiting disease, antimicrobial treatment can reduce the average duration of symptoms as well as reducing the period of *Shigella *excretion after symptoms subside [7,8]. As a result of antimicrobial use over time, *Shigella *isolates have developed resistance to many commonly used antimicrobial drugs, and mobile genetic elements including R plasmids, transposons, integrons and genomic islands on the bacterial genome can help disseminate resistance determinants [5,9-11]. Recent reports have determined the molecular basis of multidrug-resistance (MDR) phenotypes of *Shigella *spp. in Australia [12], Brazil [10], China [13], India [14], Ireland [15], Japan [16], and Korea [17]. Drug resistance patterns are influenced by many factors including geographic location, year in which the isolate was obtained, class of antimicrobial agent, pressure exerted by antimicrobial use, and isolate source [8,11].

Local knowledge of antimicrobial resistance patterns is valuable as a guide to therapy, as an epidemiologic typing method, and as an indicator of disseminating antimicrobial resistance in the region [11,18]. Specific PCR assays have been developed to screen for genes associated with virulence and antimicrobial resistance to commonly used drugs such as ampicillin, chloramphenicol, and tetracycline [5,10,19]. Plasmid profiling and chromosomal fingerprinting using pulsed-field gel electrophoresis (PFGE) are two molecular techniques that can additionally help to characterize *Shigella *isolates in endemic and outbreak situations [15,20]. Taken together, these methods provide complementary data to compare *Shigella *strains in a variety of settings and communities.

The objective of this study is to use a suite of epidemiologic and molecular characterization techniques to present a unified approach for characterizing endemic and outbreak patterns of shigellosis with a California case study example at the population level. The results described herein include case distributions, antimicrobial resistance testing, PCR for three antimicrobial resistance genes, plasmid profiling, and chromosomal fingerprinting to evaluate the type of drug resistance, risk factors associated with high levels of drug resistance, and diversity of strains present in a central California population during a two year period.

## Methods

### Shigella strains

Clinical *Shigella *isolates were obtained from the Monterey and Santa Cruz County Health Departments during 2004-2005 under the University of California, Davis Institutional Review Board Exemption Protocol 14541-1 for working with anonymous human laboratory samples. The bacteria were inoculated into Selenite-F enrichment broths and onto selective agar plates for isolation and characterization after aerobic incubation at 37°C for 24 h. *Shigella *species were identified using colony morphology and then conventional biochemical reactions including API 20E strips (Biomerieux, France) and antisera slide agglutination tests (BD Difco, MD). Serotype and demographic information was initially provided by the county health departments. The Centers for Disease Control and Prevention reference laboratory performed ipaH PCR and subserotyping of selected *Shigella *isolates, and this data was used as definitive identification in any case of discrepancies.

### Antimicrobial resistance testing

Drug resistance testing was performed using microdilution and disk diffusion methods as outlined by the Clinical Laboratory Standards Institute [21,22], and antimicrobial classes were defined as for the National Antimicrobial Resistance Monitoring System (NARMS) for enteric bacteria [23]. Sensititre Gram-negative NARMS plates (TREK diagnostic systems, OH) were used to test *Shigella *isolates for resistance to amikacin (AMK), amoxicillin-clavulanic acid (AMC), ampicillin (AMX), cefoxitin (FOX), ceftiofur (TIO), ceftriaxone (CRO), chloramphenicol (CHL), ciprofloxacin (CIP), gentamicin (GEN), kanamycin (KAN), nalidixic acid (NAL), streptomycin (STR), sulfisoxazole (FIS), tetracycline (TET), and trimethoprim/sulfamethoxazole (SXT). Inoculated Sensititre^® ^plates were incubated overnight at 35°C and examined to determine the dilution at which bacterial growth was at least 90% inhibited. For quality control, two control strains of *Escherichia coli *(ATCC 25922 and 35218) were tested as well as the internal positive and negative controls included in each microdilution plate.

Disk diffusion methods were used to test for azithromycin (AZM) resistance [22]. Mueller-Hinton agar plates were inoculated with bacteria suspended in 0.85% saline adjusted to a turbidity equivalent to a 0.5 McFarland standard and incubated with 15 μg azithromycin disks (BBL, BD Diagnostics, NJ) overnight at 37°C. The area of inhibited bacterial growth around the azithromycin disk was measured to determine whether isolates were resistant (≤ 13 mm), intermediate (14-17 mm) or sensitive (≥ 18 mm). Zone diameters for each isolate were measured manually by two investigators.

### PCR analysis

Selected *Shigella *isolates were also screened for antimicrobial resistance genes. Fresh colonies were suspended in sterile water, boiled for 10 min, and centrifuged for 10 min at 1300 rpm. The supernatant was separated from the pellet and prepared DNA was stored at -20°C. Specific primer sets and PCR protocols targeted the *tetB*, *catA *and *bla*_TEM _genes associated with resistance to tetracycline, chloramphenicol, and ampicillin, respectively [5,10]. PCR products were run on 1.3% agarose gels, stained with ethidium bromide, and photographed under UV light on a Gel Doc 2000 system (Bio-Rad, CA).

### Plasmid profiling

*Shigella *isolates were screened for the presence of plasmid DNA using standard protocols [24]. Plasmid DNA was extracted using an alkaline lysis method with a Quantum Prep Plasmid Miniprep Kit (Bio-rad), 14 μl of plasmid DNA extract was added to 4 μl of tracking buffer, and the mixture was loaded to a 0.6% agarose gel. Horizontal gel electrophoresis was carried out in 1× Tris-acetate-EDTA (TAE) buffer at 80 V for 1 h. The gel was stained with ethidium bromide and visualized under ultraviolet light (UV) using a Gel Doc 2000 system (Bio-Rad).

### PFGE analysis

Chromosomal fingerprinting using PFGE was performed on selected *Shigella *isolates according to Pulse Net protocols [25]. Bacteria were incorporated into agarose plugs, lysed, digested with XbaI restriction enzymes (New England Biolabs, MA) and then run on a 1% gel at 200 V for 18 h using a CHEF Mapper system (Bio-Rad). The electrophoresis conditions consisted of an initial switch time of 2.16 seconds and a final switch time of 54.17 seconds. The gel was stained with Gel Star (Cambrex Bioscience, Rockland, ME) and banding patterns photographed under UV light on a Gel Doc 2000 system. Cluster analysis was performed using BioNumerics software (Applied Maths Inc, Austin TX) with comparisons made using Dice coefficients and an unweighted pair-group method with arithmetic averages (UPGMA) [26].

### Data analysis

The distributions of clinical *Shigella *isolates obtained during the two year study period were grouped by month to evaluate possible endemic versus outbreak patterns in the community. Demographic data provided by the health departments were analyzed with respect to the following categorical variables: *Shigella *species (*S. flexneri, S. sonnei*), year of isolation (2004, 2005), gender (male, female), and age group (< 5 yrs, 5-18 yrs, > 18 yrs).

Antimicrobial resistance patterns were evaluated with respect to *Shigella *species, plasmid profiles, and PFGE patterns. Chi-square tests were used to compare categorical groups, and logistic regression was additionally used to evaluate risk factors in relation to the outcome of being resistant to five or more antimicrobial drugs. All analyses were performed using Stata 10.0 software (Statacorp, TX), and *P*-values < 0.1 were considered significant.

## Results

### Demographics

**Figure 1:**
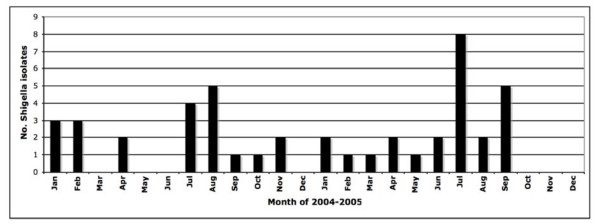
**Monthly *Shigella *distribution in the Monterey Bay region of California, 2004-2005**.

During the 2004-2005 study period, 64 *Shigella *isolates were obtained and the first isolate from each patient was included in this study, resulting in a total sample size of 43 *Shigella *isolates. Roughly half (47%) of isolates were collected in 2004 and 53% in 2005. The distribution of isolates by month is shown in Figure 1, which is consistent with an endemic level of shigellosis in the community. The highest number of monthly cases was eight in July 2005 and this group was comprised of multiple species, not a single consistent strain that would be suggestive of an outbreak. Overall, the majority (51%) of *Shigella *isolated were *S. flexneri *(serogroup B) while 49% were *S*. *sonnei *(serogroup D). Subserotyping of *S. flexneri *isolates revealed that the most common subserotypes were 3a (38% of isolates tested) and 1b (23%), followed by 1c and 2a (both 15%), and lastly a Y variant (-:3,4) that made up 7% of *S. flexneri *isolates.

**Figure 2:**
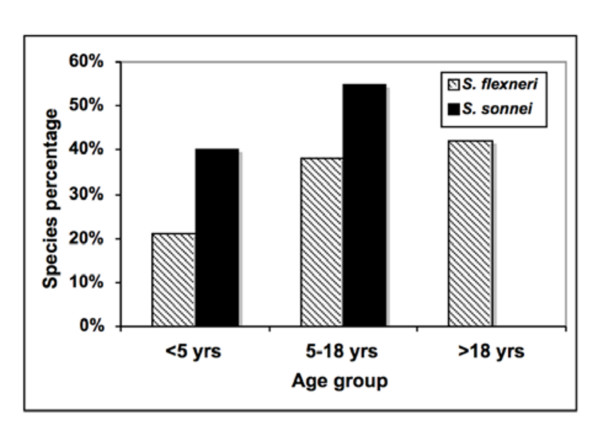
**Age distribution of *Shigella *species from the Monterey Bay region of California, 2004-2005**. *S. flexneri - Shigella flexneri; S. sonnei - Shigella sonnei*.

Among *Shigella *patients, 60% were female, 36% were male, and 4% were of unknown gender. With respect to age, 29% of *Shigella *patients were children under 5 yrs, 44% were 5-18 yrs, 24% were over 18 yrs, and 2% were of unknown age. Figure 2 shows the proportion of *S. flexneri *and *S. sonnei *within each age group. Interestingly, *Shigella flexneri *was isolated from patients in all age groups, in contrast to *S. sonnei *that was only isolated in patients under 18 yrs.

### Antimicrobial Susceptibility

**Figure 3:**
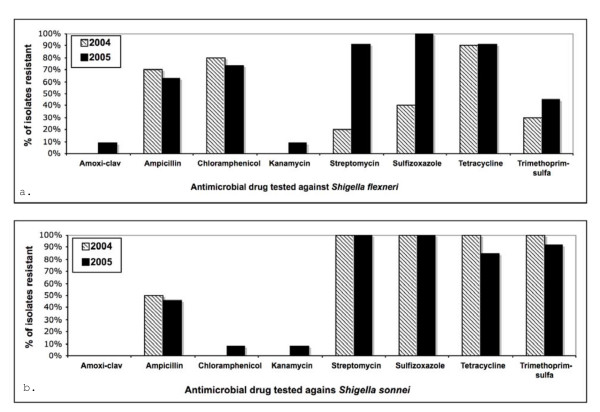
**Percent of *Shigella flexneri *(top) and *S. sonnei *(bottom) isolates fully resistant to antimicrobial drugs**. No full resistance was observed to amikacin, azithromycin, cefoxitime, ceftiofur, ceftriaxone, ciprofloxacin, gentamicin, or nalidixic acid.

Antimicrobial drug testing was carried out on 42 *Shigella *isolates divided evenly among *S. flexneri *and *S. sonnei*. Figure 3 shows the percent of *S. flexneri *and *S. sonnei *isolates fully resistant to individual antimicrobial drugs in 2004 compared to 2005. Over 60% of *S. flexneri *isolates were fully resistant to ampicillin, chloramphenicol, or tetracycline in both years. Increased resistance of *S. flexneri *to amoxicillin-clavulanic acid, kanamycin, streptomycin, sulfizoxazole, and trimethoprim-sulfa was observed in 2005 compared to 2004. For *S. sonnei*, over 80% of isolates in both years were fully resistant to streptomycin, sulfizoxazole, tetracycline, or trimethoprim-sulfa. Less than 50% of *S. sonnei *were fully resistant to ampicillin, chloramphenicol, or kanamycin. No full drug resistance in *Shigella *spp. was observed for amikacin, azithromycin, cefoxitime, ceftiofur, ceftriaxone, ciprofloxacin, gentamicin, or nalidixic acid. It is significant to note that while none of the *Shigella *isolates were fully resistant to azithromycin in this study, 86% of *S. sonnei *isolates showed intermediate resistance, as did 10% of *S. flexneri *isolates. Similarly for amoxicillin-clavulanic acid, 48% of *S. flexneri *had an intermediate level of resistance, as did 10% *S. sonnei *isolates.

**Figure 4:**
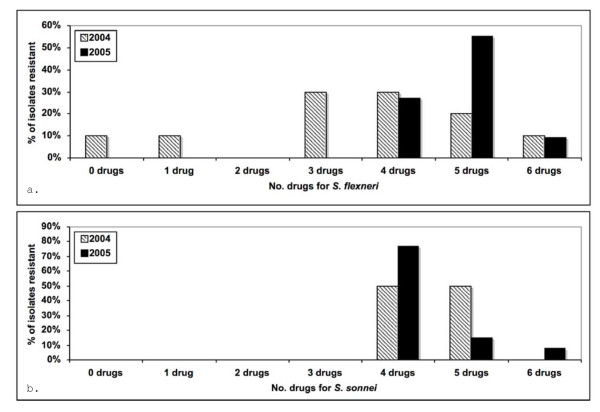
**Proportion of multi-drug resistant *Shigella flexneri *(top) and *S. sonnei *(bottom) isolates from California, 2004-2005**.

In addition to drug resistance to individual agents, multi-drug resistance is also important to consider. Over 95% of *Shigella *isolates in this study were resistant to three or more antimicrobial drug subclasses as defined by NARMS criteria and 38% of isolates were resistant to five or more. Figure 4 shows the number of drugs that *S. flexneri *and *S. sonnei *isolates were resistant to in 2004 and 2005. The majority of both species were resistant to four or more drugs in both years. The level of multi-drug resistance increased from 2004 to 2005 for *S. flexneri *isolates but the shift was less dramatic for *S. sonnei*.

Table 1 shows the drug resistance patterns (antibiograms) in this study, listed from most to least common. A third of isolates were fully resistant to streptomycin, sulfisoxazole, tetracycline, and trimethoprim-sulfa. The next most common patterns were to ampicillin, streptomycin, sulfisoxazole, tetracycline, and trimethoprim-sulfa (14% of isolates), and then to ampicillin, choramphenicol, streptomycin, sulfosoxazole, and tetracycline (10% of isolates). The highest level of resistance (six antimicrobial drugs) was detected in 7% of *Shigella *isolates and included ampicillin, chloramphenicol, streptomycin, sulfisoxazole, tetracycline, and trimethoprim-sulfa.

**Table 1:** *Shigella *drug resistance patterns with associated serotypes, plasmid profiles, and PFGE fingerprint patterns from California, 2004-2005.

Resistance pattern to the NARMS antimicrobial panel	% of isolates with resistance profile (no.)	Plasmid profile groups detected (no.)^a^	Species of isolates with plasmids (no.)	PFGE patterns
STR, FIS, TET, SXT	33% (14)	II, X(3), VIII	*S. sonnei *(4), S. *flexneri*	III, V, VI, IX, X
AMP, STR, FIS, TET, SXT	14% (6)	II(4), VI	*S. sonnei *(5)	I, IV, VII, VIII, XI, XII
AMP, CHL, STR, FIS, TET	10% (4)	none	none	
AMP, CHL, TET	7% (3)	VII	*S. flexneri *3a	XX, XXIII, XV
AMP, CHL, STR, FIS, TET, SXT	7% (3)	IV(2), V	*S. flexneri *3a (2), *S. sonnei*	
TET	5% (2)	I, IV	*S. flexneri *1b (2)	XIX
AMP, CHL, FIS, TET	5% (2)	IV(2)	*S. flexneri *2a (2)	XVII
CHL, STR, FIS, TET, SXT	5% (2)	V(2)	*S. flexneri *1c (2)	XV
AMP, CHL, FIS, TET, SXT	2% (1)	IV	*S. flexneri *2a	XVIII
AMP, KAN, STR, FIS	2% (1)	IV	*S. sonnei*	XIII
CHL, FIS, TET, SXT	2% (1)	III	*S. flexneri *Y var.	XXIV
AMP, CHL, STR, TET	2% (1)	IX	*S. flexneri *1b	XIV
AMP, STR, FIS, SXT	2% (1)	IX	*S. sonnei*	
AMC, AMP, GEN, STR, FIS	2% (1)	IV	*S. flexneri*	

^a ^Of the 42 isolates tested, 62% (n = 26) had plasmids detected while 38% (n = 16) did not.

Logistic regression analysis identified two risk factors statistically associated with high drug resistance as defined by full resistance to five or more drugs. In the univariate analysis, high drug resistance was more likely to be associated with *S. flexneri *than *S. sonnei *(*P *= 0.06), and more likely to be detected in female than male patients (*P *= 0.07). Age group and year of isolation were not significant risk factors. However, when both *Shigella *species and patient gender were included in the regression model, *S. flexneri *species was positively associated with high drug resistance (*P *= 0.09), while gender was not significant (*P *= 0.12). In the final model, high drug resistance was 1.2 times more likely in *S. flexneri *than *S. sonnei*, and other risk factors were not significant.

### PCR analysis

Molecular PCR amplification resulted in gels with the 415, 457, and 559 bp bands expected for genes associated with tetracycline (*tetB*), chloramphenicol (*catA*), and ampicillin (*bla*_TEM_) resistance, respectively. Of the 33 tetracycline-resistant *Shigella *tested, 100% (16/16) of *S. flexneri *and 71% (12/17) of *S. sonnei *were positive for the *tetB *gene. Of the 14 chloramphenicol resistant isolates tested, 85% (11/13) of *S. flexneri *and 100% (1/1) *S. sonnei *were positive for the *catA *gene. Of the 24 ampicillin resistant *Shigella *tested, 21% (3/14) of *S. flexneri *and 44% (4/9) of *S. sonnei *were positive for the *bla*_*TEM *_gene.

### Plasmid profiling

Ten different plasmid profiles (I-X) were detected in this study, with plasmid bands ranging from 1.5-10 kb in size. Of the 42 isolates tested, 62% has plasmids and 38% did not. Profile I had 1.5 and 2 kb plasmids, profile II had 1.5, 4, and 5 kb plasmids, profile III had 1.5, 2, 3, 5, 6, and 10 kb plasmids, profile IV had 2 and 2.5 kb plasmids, profile V had 1.5, 2, and 3 kb plasmids, profile VI had 1.5 and 3 kb plasmids, profile VII had 1.4, 2, 2, 5, and 3 kb plasmids, profile VIII had 2 and 4.5 kb plasmids, profile IX had 1.5, 3, and 4 kb plasmids, and profile X had 1.5, 3, 4, and 5 kb plasmids. Table 1 shows the distribution of plasmid profiles and serotypes by antibiogram group, with some antibiogram groups including multiple plasmid profiles and serotypes.

### PFGE

**Figure 5:**
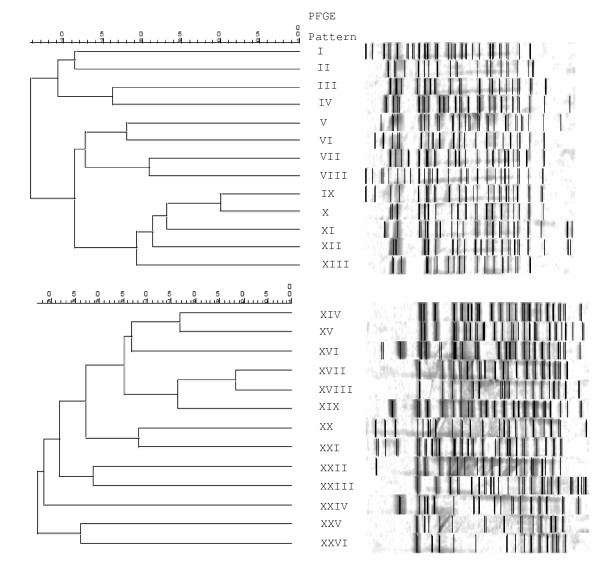
***Shigella sonnei *(top) and *S. flexneri *(bottom) dendrograms showing intra-species relatedness of Pulsed Field Gel Elecrophoresis (PFGE) fingerprints after XbaI digestion**.

Twenty-six *Shigella *isolates were selected for PFGE fingerprinting, with an even number of *S. sonnei *and *S. flexneri *represented, and at least one isolate from each plasmid profile included in the chromosomal fingerprinting analysis. The PFGE patterns after XbaI digestion are shown in Figure 5 as dendrograms of genetic intra-species relatedness. All PFGE patterns were unique and thus show that there is a high level of diversity in *Shigella *from the Monterey region of California. This provides molecular evidence to support the endemic epidemiologic pattern in time that was observed in the initial epidemiologic evaluation (Figure 1). The distribution of PFGE patterns by antibiogram group and in relation to plasmid profile groups and serotypes are shown in Table 1, again highlighting the diversity of endemic strains in this population.

## Discussion

This study shows how epidemiologic patterns at the host and bacteria population levels can be used to investigate endemic as compared to outbreak patterns of shigellosis in a community. The observed pattern in time, with a small number of cases coming in throughout the year, along with the high diversity in molecular fingerprints of *Shigella *isolates support an endemic pattern in this population. A true spatial analysis would also be useful to evaluate clustering and additional risk factors for infection, but due to patient confidentiality that data was not available. Traditional and molecular epidemiologic data gathered as part of public health investigations will be instrumental in identifying emerging antimicrobial resistance, developing treatment guidelines appropriate for communities, and providing baseline data with which to compare future outbreak strains.

There is worldwide concern about the emergence of drug resistant strains of enteric pathogens. In this study, we found that over 95% of *Shigella *isolates from the Monterey region of California were resistant to three or more antimicrobial drug classes, and 38% were resistant to five or more. This level of drug resistance is higher than national NARMS data for the same 2004 time period, which reported 62% resistance to three or more drug subclasses, and 28% resistance to five or more [23].

Another difference between this central California study and national data is that in this study *S. flexneri *was the more prevalent species, followed closely by *S. sonnei*. Most studies have reported *S. sonnei *to be the most common species in developed countries such as the United States, with *S. flexneri *more prevalent in developing countries [1-4,27,28]. For example, the Shiferaw [28] study reported that *S. sonnei *accounted for 70% of *Shigella *infections in the United States, followed by *S. flexneri *associated with 24% of infections. In contrast, we found that in the Monterey region of California, *S. flexneri *accounted for 51% of isolates, followed by *S. sonnei *at 49%. Additionally, we found that *S. flexneri *types 3a and 1b were most common, followed by 1c, 2a, and a Y variant (-:3,4). Subserotypes 2a and 3a have been reported as common in studies from India, Thailand, and Malaysia, while subserotype 1b was the predominant type in studies from Malaysia and Peru [4]. A 2007 study [29] of adults in San Francisco, CA conducted during 1998-1999 found a similar species distribution to our study, with *S. flexneri *making up 53% of isolates and *S. sonnei *46%, while a previous 1996 study [27] that included all age groups found the more typical distribution of *S. sonnei *predominating over *S. flexneri *at 62% versus 32%, respectively. No subserotyping data was available from the San Francisco studies.

The majority of shigellosis cases in our study occurred in children. Approximately 40% of *S. sonnei *isolates were detected in children under 5 years old, with the other 60% of isolates detected in youth 5-18 years old. *Shigella flexneri *isolates were more evenly distributed among all age groups, with 20% of isolates occurring in children under 5 years, 40% in youth 5-18 years, and 40% in adults over 18 years old. Most other studies have reported the majority of shigellosis cases in children [8,11,28,30] though the Baer et al. study [27] in San Francisco found the majority of cases in adults over 18 years old, along with the risk factors of male gender, white ethnicity, and being HIV-positive.

Ethnicity has been associated with *Shigella *infection in the United States [28-31]. The Shiferaw et al. [28] study that utilized the Foodborne Diseases Active Surveillance Network (FoodNet) reported a higher incidence of *S. sonnei *and *S. flexneri *cases among patients of black and Hispanic ethnicity compared to white patients. That study also noted that the highest incidences of shigellosis in the country occurred in California and Georgia. The Kalluri et al. [31] study of *S. boydii *epidemiology reported that the majority of patients were Hispanic, and that most of the patients who had traveled abroad in the week before symptoms occurred had gone to Mexico. In our study we did not have ethnicity data linked to individual cases, but we can speculate that our results could be similarly affected given the high percentage of Hispanics with shigellosis (approximately 86% of cases from 2003-2007) in the Monterey region [32]. Many are migrant workers who travel regularly to their countries, so it is plausible that *Shigella *strains are being imported with these travelers. The high prevalence of *S. flexneri *could also reflect strains circulating among subgroups such as homosexual populations, as reported in San Francisco [29].

Treatment of shigellosis should be guided by local antimicrobial resistance patterns. In this central California study, we found no full resistance to amikacin, azithromycin, cefoxitime, ceftiofur, ceftriaxone, ciprofloxacin, gentamicin, and nalidixic acid. However, significant intermediate resistance was observed to azithromycin in *S. sonnei *isolates, and to amoxicillin-clavulanic acid in *S. flexneri *isolates. The results indicate that *Shigella *species could develop full resistance to these two drugs in the future, and this should be considered when prescribing them as empirical therapy for shigellosis. Importantly, we observed full resistance to many drugs commonly used for shigellosis treatment, with high resistance noted for ampicillin, chloramphenicol, streptomycin, sulfisoxazole, tetracycline, and trimethoprim-sulfa. This finding is concerning but not surprising in light of the Sur et al. [11] review that discusses how drug resistance has developed in the same order in which new drugs for shigellosis treatment were introduced. In the 1940's, tetracycline and then chloramphenicol were recommended for treatment when sulphonamides became ineffective. When further drug resistance developed, ampicillin and co-trimoxazole were recommended, and by the 1980's, some *Shigella *strains were resistant to all of these drugs but susceptible to the newly introduced fluoroquinolones. In recent studies, drug resistance has been observed to fluoroquinolones as well [33,34].

The prevalence of resistance to individual drugs in our study was somewhat different than the NARMS data for the 2004 year. *Shigella flexneri *isolates in this California study showed comparable levels of resistance to the national level data, but *S. sonnei *isolates showed more resistance to streptomycin, sulfisoxazole, tetracycline, and trimethoprim-sulfa and less resistance to ampicillin than the national data. The almost 100% of *S. sonnei *isolates that were fully resistant to streptomycin, sulfisoxazole, tetracycline, and trimethoprim-sulfa in the California study, compared to roughly 50% of isolates found resistant in the NARMS national report, is more consistent with what you might expect in developing countries. The high level of resistance in the Monterey region could have developed in response to local use of these four antimicrobials for shigellosis treatment, or the resistant strains might have been imported from developing countries via the traveling portion of the population.

Interpretation of the disk diffusion method for azithromycin can be challenging due to the double zone of inhibition observed for many *S. sonnei *isolates. Unlike most antimicrobial disk testing, azithromycin testing of *Shigella *isolates can produce an inner zone of complete growth inhibition around the disk, surrounded by an outer zone of reduced growth [35]. This double zone phenomenon was observed in our study also, mainly among *S*.*sonnei *but also among a few *S*.*flexneri *isolates. In order to make an accurate and consistent interpretation of the zone diameter, it is recommended to only measure the inner zone of complete growth inhibition to interpret antimicrobial susceptibility [35]. Using the diameter of the outer zone of reduced growth to interpret antimicrobial susceptibility could result in underestimating the level of resistance.

A variety of mechanisms may be responsible for the development of antimicrobial resistance among *Shigella *strains. Resistance may spread clonally during an outbreak situation or horizontally by plasmids, transposon-mediated conjugation, or chromosomal gene transfer [11]. In the current study we screened a subset of *Shigella *isolates for genes associated with antimicrobial resistance to ampicillin, chloramphenicol, and tetracycline, and found that some but not all isolates in each antimicrobial resistance category carried the target genes. Our results are consistent with the Toro et al. [5] and Navia et al. [9] studies that found a heterogeneous distribution of resistance determinants among *S. flexneri *and *S. sonnei *isolates in Chile and Tanzania, respectively. Additional molecular studies could be performed to further characterize drug resistance and virulence alleles present in study isolates.

Plasmid profiling and chromosomal fingerprinting both showed a high level of diversity among *Shigella *from the Monterey Bay region of California. Of the 42 isolates characterized by plasmid profiling, 62% contained plasmids and these were distributed among ten different profiles. Of the 26 isolates characterized by PFGE, all produced unique chromosomal fingerprint patterns. The high level of diversity present in the *Shigella *population suggests an endemic as compared to outbreak scenario in this community, and that this suite of molecular techniques will be useful in distinguishing outbreak from endemic strains in California in the future. Indeed, other studies have used plasmid profiling and PFGE to investigate the molecular epidemiology of shigellosis in a variety of settings worldwide [9,15,19,20].

## Conclusion

This study showed how epidemiologic patterns at the host and bacterial population levels can be used to investigate endemic as compared to outbreak patterns of shigellosis in a community. Information gathered as part of such investigations will be instrumental in identifying emerging antimicrobial resistance, for developing treatment guidelines appropriate for that community, and to provide baseline data with which to compare outbreak strains in the future. This study confirms findings from other parts of the world that point to a continued emergence of multi-drug resistant strains of enteric pathogens in the face of widespread antimicrobial use.

## Competing interests

The authors declare that they have no competing interests.

## Authors' contributions

SA was involved in all parts of the study, with a main focus on the molecular characterization studies and initial manuscript preparation. WM conceived of the study, trained SA, guided the study design and analysis, and prepared the manuscript for publication. BB provided expertise and facilities for antimicrobial resistance testing, and contributed to manuscript preparation. GG provided all isolates and contributed to manuscript preparation. LC provided expertise and facilities for the molecular characterization studies, and contributed to manuscript preparation. All authors read and approved the final manuscript.

## Authors' Information

SA was a Master's student under the co-mentorship of WAM and LC. WAM is adjunct faculty at the School of Veterinary Medicine (SVM), University of California Davis (UCD) and has expertise in epidemiology and microbiology. BB is a faculty microbiologist at SVM, UCD with extensive clinical microbiology experience. GG is laboratory director at the Monterey County Public Health Department and has extensive microbiology experience. LC is a faculty microbiologist at San Francisco State University.

## Pre-publication history

The pre-publication history for this paper can be accessed here:

http://www.biomedcentral.com/1471-2334/9/184/prepub
